# Touch Empowerment: Self‐Sustaining e‐Tattoo Thermoelectric System for Temperature Mapping

**DOI:** 10.1002/advs.202403775

**Published:** 2024-12-26

**Authors:** M. A. S. Almeida, A. L. Pires, J. L. Ramirez, S. B. Malik, S. de la Flor, E. Llobet, A. T. Pereira, A. M. Pereira

**Affiliations:** ^1^ IFIMUP Physics for Advanced Materials, Nanotechnology and Photonics, Department of Physics and Astronomy, Faculty of Sciences University of Porto Rua do Campo Alegre Porto 4169‐007 Portugal; ^2^ MINOS Universitat Rovira i Virgili Avda. Països Catalans, 26 Tarragona 43007 Spain; ^3^ Department of Mechanical Engineering Universitat Rovira i Virgili Avda. Països Catalans, 26 Tarragona 43007 Spain; ^4^ i3S Institute for Research and Innovation in Health Rua Alfredo Allen 208 Porto 4200‐135 Portugal

**Keywords:** e‐tattoo, flexible sensor, infection detection, numerical simulation, screen‐printing, temperature mapping, thermoelectric sensor, wearable sensor

## Abstract

In recent advancements within sensing technology, driven by the Internet of Things (IoT), significant impacts are observed on health sector applications, notably through wearable electronics like electronic tattoos (e‐tattoos). These e‐tattoos, designed for direct contact with the skin, facilitate precise monitoring of vital physiological parameters, including body heat, a critical indicator for conditions such as inflammation and infection. Monitoring these indicators can be crucial for early detection of chronic conditions, steering toward proactive healthcare management. This study delves into a thermoelectric sensor e‐tattoo designed for detailed skin temperature mapping. Utilizing a novel design, this sensor detects temperature variations across thermoelectric stripes, leveraging screen‐printed films of p‐type Bi_0.35_Sb_1.65_Te_3_, n‐type Bi_2_Te_2.8_Se_0.2_, and poly(vinyl alcohol) (PVA) for enhanced thermoelectric and flexible properties. The application of a prototype printed thermoelectric device on temporary tattoo paper, a pioneering development in wearable health technology is demonstrated. This device, validated through numerical simulations, exhibits significant potential as a non‐invasive tool for temperature monitoring, highlighting its value in health diagnostics and management.

## Graphical abstract

This study presents the development of a thermoelectric sensor in the form of an electronic tattoo, designed to monitor skin temperature/touch at multiple points. Using screen‐printed films of p‐ and n‐type thermoelectric materials, it demonstrates high temperature/touch sensitivity. A prototype on temporary tattoo paper shows potential for wearable health monitoring.

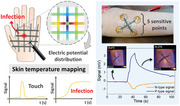

## 1 Introduction

In an era marked by the escalating importance of data, environmental stewardship, and individual health monitoring, the integration of wearable technologies has assumed a central role in contemporary society. These technologies, epitomized by a diverse array of smart wearables, fulfill a critical function in the partial surveillance of crucial parameters in our daily lives. This burgeoning trend signals the embryonic stages of the Internet of Things (IoT), a paradigm underpinned by the rapid transmission of data, most notably exemplified by the advent of the fifth‐generation of the Internet (5G). IoT, as a technological landscape, has the potential to revolutionize the way we interact with our surroundings and the information it generates. The foundation of IoT is the interconnectedness of devices, sensors, and systems, all communicating and exchanging data in real‐time. This interconnectedness has vast implications for environmental monitoring, health management, and data analysis.^[^ ^1^ ^]^

In the past decade, flexible electronics have emerged, facilitating the development of wearable electronics to enhance contact with the human body, improve measurement accuracy, and enable continuous monitoring.^[^ ^2^, ^3^, ^4^ ^]^ These devices require high sensitivity, resolution, flexibility, and portability, as well as the use of straightforward and scalable printing methods. The integration of wearable electronics with IoT opens up the possibility of continuous patient monitoring, along with the capability to intervene efficiently when necessary.^[^ ^5^ ^]^

Acknowledging the established significance of thermal fluctuations as potential indicators of inflammation and infection, the ongoing monitoring and detection of these variations might provide an early indicator of chronic conditions preceding observable superficial changes in human tissues.^[^ ^6^, ^7^ ^]^ One of the contexts in which temperature is a reliable indicator of inflammation is the skin's surface due to increased blood flow. This phenomenon is notable in processes such as wound healing or ulcerations, where localized temperature fluctuations can reach up to 2.2 K.^[^ ^7^, ^8^ ^]^ Currently, conventional devices employed in the field of biomedicine for detecting temperature variations typically rely on the use of infrared digital cameras or single‐point temperature sensors. However, they come with limitations, including the need to immobilize the patient and the inability to perform the temperature mapping of the skin.^[^ ^9^ ^]^ To overcome these challenges, researchers have explored non‐invasive wearable sensors for continuous monitoring of the skin surface, including resistive sensors^[^ ^7^, ^9^, ^10^ ^]^ and thermoelectric (TE) sensors.^[^ ^11^, ^12^, ^13^, ^14^, ^15^ ^]^ Noteworthy findings regarding these sensors are presented in **Table** **1**.

**Table 1:** Features of wearable sensors found in the literature with their respective thermal sensitivities. Depending on the involved phenomenon, these sensors can be categorized into three different groups, namely resistive sensors, horizontal thermoelectric sensors, and vertical thermoelectric sensors.

Phenomenon	Materials	Deposition method	Sensitivity	Ref.
Resistive Local absolute temperature	PDMS, PI and Cu	Spin coating, evaporation and photolithography	79 Ω K^−1^	[7]
	PDMS, CNT and PEDOT:PSS	Screen‐printing	0.25% K^−1^	[10]
	Au	Microlithographic techniques	——	[16]
	Ag nanowires	Spray coating, spin coating and laser cutting	0.47 Ω K^−1^	[17]
Thermoelectric Vertical sensors	PEDOT:PSS and PU	Immersion	32.8 mV K^−1^	[11]
	CNT/PEDOT:PSS/ nanocellulose	Freeze‐drying and thermal processing	30.5 µV K^−1^	[18]
	Cu, Ag and n‐type and p‐type Bi_2_Te_3_	Multisteps (spin coating, peel‐off and soldering)	∼19 mV K^−1^	[12]
Thermoelectric Horizontal sensors	Bi_2_Te_2.7_Se_0.3_ and Sb_2_Te_3_ with epoxy, MHHPA and EMIP	Screen‐printing	∼3 mV K^−1^	[13]
	Ag and PEDOT:PSS/LiTFSI	Screen‐printing	∼25 µV K^−1^	[14]
	PEDOT:PSS/CNT/WPU and Cu	Molding	∼31 µV K^−1^	[15]

PDMS = Polydimethylsiloxane; PI = Polyimide; CNT = Carbon nanotubes; PEDOT:PSS = Poly(3,4‐ethylenedioxythiophene) ‐poly(styrenesulfonate); PU = Polyurethane; MHHPA = Methylhexahydrophthalic anhydride; EMIP = 2‐ethyl‐4‐methyl‐1H‐imidazole‐1‐propanenitrile; LiTFSI = Lithium bis(trifuoromethanesulfonyl)imide; WPU = Waterborne polyurethane.

Resistive sensors are commonly fabricated using materials with temperature‐dependent resistance, including Copper (Cu), Gold (Au), Carbon Nanotubes (CNT), and Poly(3,4‐ethylenedioxythiophene):poly(styrenesulfonate) (PEDOT:PSS). For instance, Hattori et al.^[^ ^7^ ^]^ designed a stretchable, conformal, multifunctional electronic sensor for precise quantitative measurements relevant to cutaneous wound management. They used materials such as silicone, polyamide, and Cu, that match human skin, and integrated a set of micro‐metal resistors to enable high‐precision multimodal measurements of skin temperature and thermal conductivity after disinfected. However, a persistent issue arises from the emergence of cracks caused by dermal movement, resulting in resistive fluctuations independent of temperature variations. Recently, stretchable sensors composed of silver (Ag) nanowires for strain and temperature measurement emerged, offering no cross‐talk^[^ ^17^ ^]^ and minimal humidity influence.^[^ ^19^ ^]^ Nevertheless, all the developed sensors are designed for single‐point analysis. Since infections are often identified by temperature gradients, with the hottest point marking the origin of the infection, multiple resistive sensors capable of simultaneous measurements are required. Consequently, the practical deployment of these sensors often demands the integration of complex measurement circuits. A notable example is the work by Shin,^[^ ^20^ ^]^ where the author presented an array‐like sensor with 25 measurement points but also 25 individual signals to analyze, which can be impractical for real‐world applications. In contrast, TE sensors capitalize on the Seebeck effect inherent in their materials, a phenomenon unaffected by the sample's physical structure or resistive changes. The temperature gradient (*ΔT*) induces an asymmetry in the material's charge distribution, generating a potential difference (ΔV) determined by the material's Seebeck coefficient (S).^[^ ^21^ ^]^ This coefficient is expressed as *S* =  −Δ*V*/Δ*T*,^[^ ^22^, ^23^, ^24^ ^]^ thereby simplifying the process of signal acquisition when compared to resistive sensors. Two discrete typologies of TE sensors can be delineated upon their configuration: the vertical and horizontal variants (see Table 1). Numerous studies harness the TE phenomenon to the development of temperature sensors. For instance, Zhang et al.^[^ ^11^ ^]^ created a TE sensor to analyze the temperature difference between the skin surface and the external environment, specifically, a vertical *ΔT*. This sensor was composed of PEDOT:PSS and PU, exhibiting a sensitivity of 32.8 mV K^−1^. While these sensors are good for detecting touch and external temperature variations, they are less effective in monitoring horizontal temperature gradients. As a result, different configurations are employed to analyze these horizontal gradients and enable their application in infection monitoring. One of the examples is the configuration proposed by Manoj Jose et al.,^[^ ^14^ ^]^ which employs a single TE stripe with a sensitivity of 25 µV K^−1^. In this configuration, one end of the stripe contacts the wound, while the other end is placed in a localization with a stable temperature away from the infection. Citing this example to showcase the viability of this sensing approach also unveils a pertinent challenge: the inability to assess temperature gradients in the central region of the TE material. The analysis is constrained solely to the periphery of the stripes, rendering precise alignment over infection sites unpredictable. Moreover, employing this technique for infection detection might entail a significant number of stripes and subsequent signal analyses for temperature mapping. Hence, optimizing the ratio between signals and sensitive points is essential to simplify sensor integration.

In addition to their ability to detect temperature gradients independently of form factor, TE materials have been explored as wearable micro‐energy generators, converting human body heat into electrical energy.^[^ ^3^, ^25^, ^26^, ^27^, ^28^ ^]^ Through this functionality, it becomes possible to create self‐powered TE sensors, that do not rely on traditional external power sources.^[^ ^26^, ^29^ ^]^ While for sensing purposes, it is primarily important to determine the *S*, for power generation, factors such as the electrical conductivity, σ, and thermal conductivity, κ, of the TE materials must also be considered. This allows reducing internal resistive losses and enhances the ability to maintain the *ΔT*. The overall efficiency can be given by the Figure of Merit, *ZT*, or the Power Factor, *PF*.^[^ ^23^ ^]^

Currently, the best‐performing materials for TE applications at room temperature are rigid inorganic materials, such as Bi_2_Te_3_, which is renowned for having the highest *ZT*, along with PbTe, SiGe, among others.^[^ ^29^, ^30^ ^]^ However, to adapt these materials for use in flexible electronics, their micro and nanoparticles have been combined with flexible polymers to create printable TE inks, resulting in hybrid TE materials.^[^ ^4^, ^24^, ^31^, ^32^ ^]^ These hybrid materials not only offer flexibility but also boast attributes like cost‐effectiveness, low mass density, abundance, low thermal conductivity, and ease of manufacture through straightforward deposition techniques, including screen printing, inkjet printing, and spin coating, among others.^[^ ^33^ ^]^ To formulate the printable TE inks, conductive polymers such as PEDOT:PSS,^[^ ^34^ ^]^ Polyaniline (PANI),^[^ ^35^ ^]^ and Polypyrrole (PPY)^[^ ^36^ ^]^ are commonly employed. Additionally, findings using insulating polymers like Poly(Vinyl Alcohol) (PVA),^[^ ^37^ ^]^ Polyvinylidene fluoride (PVDF) and Poly(methyl methacrylate) (PMMA)^[^ ^38^ ^]^ have been reported. For instance, Gao et al.^[^ ^18^ ^]^ also propose a high‐resolution temperature sensor based on a laminar TE aerogel of CNT/PEDOT:PSS/nanocellulose. Their aerogel exhibits high sensitivity (30.5 µV K^−1^) and a resolution of 0.02 K. In this context, it is very important to ensure the percolation within the printable films.

**Figure 1:**
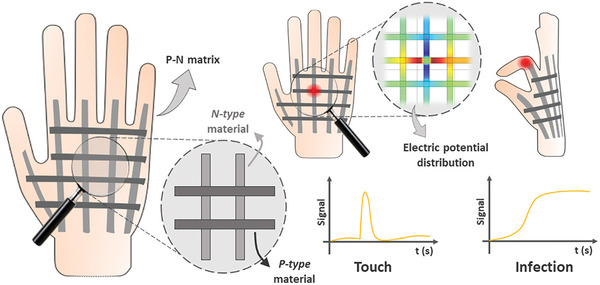
Scheme of the thermoelectric configuration proposed in the current study for the detection of horizontal temperature gradients on the skin's surface. This configuration comprises p‐type and n‐type thermoelectric stripes transverse to each other, creating sensitivity points. Temperature fluctuations within the stripes lead to changes in the potential electrical distribution across the network, facilitating the location of these changes. Signal analysis enables differentiation between physical contact (touch) and infections.

A configuration of TE materials is presented herein and tailored to enable comprehensive skin surface temperature mapping with reduced contact points and electrical signal inputs. The primary objective is to forge new avenues for the integration of TE sensors. This sensor design incorporates transversely aligned p‐type and n‐type TE stripes, strategically positioning measurement nodes at their termini. This layout engenders a grid‐like array of sensitivity nodes at the intersections and terminations of the stripes, proficient in discerning temperature fluctuations prompted by infections or external stimuli. Through a nuanced analysis of signal waveforms and potential electrical distributions across this grid, real‐time detection, localization, and differentiation of infections and external contacts become feasible. This capability endures even under strain in flexible TE materials, as depicted in **Figure** **1**. Importantly, this configuration distinguishes itself from extant literature by enabling the assessment of lateral temperature gradients across multiple points within the sensor plane. Furthermore, it obviates the necessity for highly precise deposition techniques, presenting a simplistic geometry adaptable for deployment across various regions of the human body.

In the first stage of this work, our focus is on optimizing the TE and flexible properties inherent in the chosen materials. This is followed by outlining the conceptual framework of our proposed configuration, a conceptualization supported by both numerical simulations and empirical validation. To demonstrate the feasibility of our approach, we employ a screen‐printing process to create a TE e‐tattoo, serving as a tangible proof‐of‐concept prototype for subsequent rigorous testing.

## 2 Thermoelectric Printed Films: Optimization and Characterization

The first step for the production of a TE e‐tattoo is the optimization of TE films. The production of flexible printed films began by blending commercial TE powders with PVA doped with H_3_PO_4_.^[^ ^29^ ^]^ The composition of the TE powders was determined by (SEM) and X‐Ray Diffraction (XRD) to be Bi_0.35_Sb_1.65_Te_3_ and Bi_2_Te_2.8_Se_0.2_ for the p‐type and n‐type material, respectively. In this way, through this work will be denominated as p‐BiSbTe and n‐BiTeSe. All the characterization of these commercial powders is presented in Appendix A from the supporting information document (see Figures S–A1, S–A2, and Table S–A1, Supporting Information). Through a deliberate adjustment of inorganic material concentrations, ranging from 19 to 47 Vol.%, the TE ink was optimized to suit screen printing techniques. After applying these distinct ink formulations onto a pre‐cleaned flexible polyimide substrate using the screen‐printing method, a thorough analysis was carried out to investigate their structural, morphological, and transport properties.

**Figure 2:**
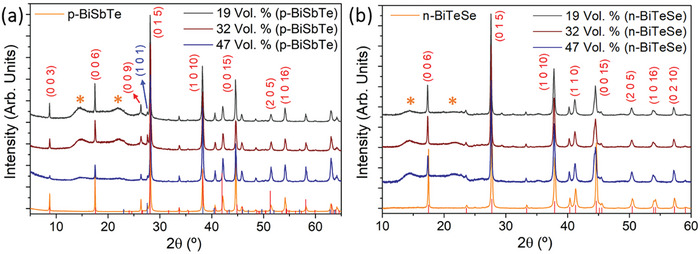
X‐ray diffraction pattern of the produced printed films with inorganic concentrations of 19, 32, and 47 Vol.%, namely for a) p‐type films with identification of the peaks belonging to the crystalline phase of Bi_0.35_Sb_1.65_Te_3_ (vertical red lines) and Te (vertical blue lines), and b) n‐type films with identification of the peaks belonging to the crystalline phase of Bi_2_Te_2.8_Se_0.2_ (vertical red lines). (Note: Soft peaks identified with * are attributed to the Kapton substrate.).

The X‐ray diffractograms presented in **Figure** **2**a,b portray the results obtained from the printed films generated in this study. These figures confirm the polycrystalline nature of both the p‐BiSbTe and n‐BiTeSe TE printed films. No discernible alterations are observed when compared with the diffraction patterns of the original powders. Moreover, within the angular range of 2θ = 15° to 25°, two additional smooth peaks are evident, aligning distinctly with the Kapton diffraction pattern.

**Figure 3:**
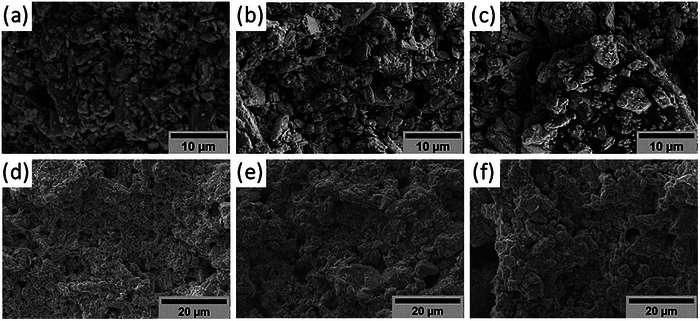
SEM images of the printed films with a) 47, b) 32 and c) 19 Vol% of p‐BiSbTe powder, and d) 47, e) 32 and f) 19 Vol% of n‐BiTeSe powder.

In **Figure** **3**, the SEM analysis illustrates surface images of the printed films developed in this study. These visuals reveal the random distribution of TE particles within the polymer matrix without considerable change in the average diameter compared with powder. Notably, films with higher polymer concentrations (Figure 3c,f) exhibit a more prominent presence of the polymer, evidenced by a discernible darker layer atop the particles. In contrast, films with lower polymer concentrations (Figure 3a,d) display higher particle compaction.

**Figure 4:**
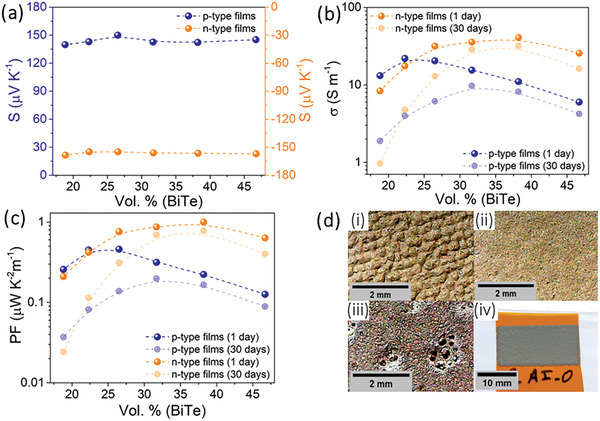
Transport properties of the thermoelectric films produced using PVA/H_3_PO_4_. a) Seebeck coefficient, ***S***; b) Electrical conductivity, σ; c) Power Factor, ***PF***; d) Surface images of produced films: Microscope image of the film with i) 47 Vol.% (p‐BiSbTe), ii) 32 Vol.% (p‐BiSbTe) and iii) 19 Vol% (n‐BiTeSe), and a complete image of the film with 32 Vol.% (p‐BiSbTe).

**Figure** **4** shows the transport properties of the printed films. Across all variations in concentration, the *S* remains consistent and equal to 145 ± 5 µV K^−1^ and −154 ± 4 µV K^−1^ to the p‐type and n‐type TE materials, respectively (depicted in Figure 4a). These values closely resemble those of the original powders, indicating minimal influence from the organic constituents. This marginal impact is attributed to the polymer's low *σ*, allowing the predominant *S* to originate from the inorganic content.^[^ ^18^ ^]^ This finding strongly aligns with the research goals, emphasizing the direct correlation between sensor sensitivity and the *S*.

Unlike the *S*, the *σ* of the fabricated films exhibits dependence on the volumetric concentration of the material. Moreover, a time‐dependent analysis (aging) of *σ* was conducted for all printed films, examining two time points: 1 day and 30 days post‐printing, as illustrated in Figure 4b. For the first case, comparing these films to the original powder (*σ* = 119 S cm^−1^ for n‐BiTeSe and *σ* = 116 S cm^−1^ for p‐BiSbTe, see Supporting Information), a drastic reduction up to three orders of magnitude in the *σ* is evident. This significant decrease is attributed to the incorporation of a poorly electrically conductive material employed in producing the printed films. Beyond the use of this material with low *σ*, variations in the printing quality of the films may also contribute to the observed changes in *σ*. The optical microscope analysis (Figure 4d) established a link between print quality and *σ*. Higher inorganic material concentrations led to high viscosity, resulting in rough surfaces and low *σ*. Increasing polymer content decreased viscosity, yielding smoother films with small pores and higher *σ*. Lower inorganic material concentrations produced films with large pores, reducing *σ*. Optimized concentrations differed for p‐type and n‐type films due to varying particle size dispersion, with 22 Vol.% (p‐BiSbTe) and 32–38 Vol.% (n‐BiTeSe) as the respective optimal concentrations for continuous film production. The films’ good uniformity of the entire length was also confirmed for the intermediate concentrations by measuring the thickness of the films at different points. For example, the thickness of the films with 32 Vol.% (n‐BiTeSe) and 32 Vol.% (p‐BiTeSe) were 57 ± 4 µm and 67 ± 5 µm, respectively.

The temporal stability of σ emerges as a crucial parameter, evaluated 30 days post‐printing and displayed as the most transparent color in Figure 4b. Notably, both p‐type and n‐type films with higher proportions of inorganic material exhibit a pronounced decrease in *σ*, sometimes up to 10 times smaller. Specifically, films with 38 Vol.% (BiTe) demonstrate better stability, retaining ≈78% of their initial values. This decline in *σ* can be largely attributed to the degradation of the organic material over time.

Figure 4c depicts the trend of the PF for the films produced in this work. From that figure, it is evident that the PF follows the behavior of the *σ*. The most efficient films, one day after printing, are those with 22 Vol.% (p‐BiSbTe) with σ = 21.97 S m^−1^ and PF = 0.45 µW K^−2^ m^−1^, and with 38 Vol.% (n‐BiTeSe) with σ = 40.8 S m^−1^ and PF = 1.00 µW K^−2^ m^−1^. Thirty days after printing, due to thin film degradation, the most efficient films become 32 Vol% (p‐BiSbTe) with 45% of this best initial PF, and 38 Vol.% (n‐BiTeSe) with 78%.

Our findings illuminate the strategic utilization of a doped insulating polymer, notable for its superior *S*. Moreover, our approach, marrying the application of widely available materials with an efficient, scalable printing process amenable to roll‐to‐roll production, signifies a pivotal enhancement in the fabrication methodologies of TE devices. This advancement underscores the potential for expanded practical application and the pathway toward commercialization in the field.

Although the TE efficiency attained in our study does not match the higher benchmarks set by research involving conductive polymers such as PEDOT:PSS,^[^ ^34^, ^39^, ^40^ ^]^ our findings illuminate the strategic utilization of a doped insulating polymer, notable for its superior *S*. Moreover, our approach, marrying the application of widely available materials with an efficient, scalable printing process amenable to roll‐to‐roll production, signifies a pivotal enhancement in the fabrication methodologies of TE devices.^[^ ^41^, ^42^ ^]^ The values achieved in this work closely align with those reported for the mixture between PANI and Bi_2_Te_3_,^[^ ^43^ ^]^ and are higher than those previously reported by our group for Bi_2_Te_3_ and PVA/H_3_PO_4_ printed by stencil printing.^[^ ^44^ ^]^

**Figure 5:**
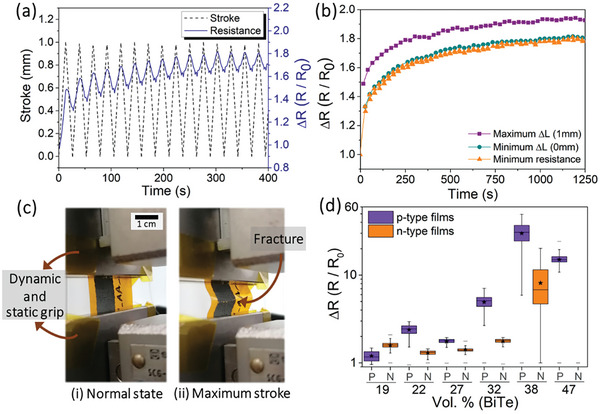
Flexibility analysis of the samples produced. a) Resistance variation in the first 16 cycles performed with the sample with 32 Vol.% (n‐BiTeSe); b) Resistive variation values at the point of maximum deformation, minimum deformation and minimum resistance during the 50 measurement cycles; c) Image of the sample with 47 Vol.% (p‐BiSbTe) in the relaxed state and with maximum deformation, where it is possible to observe a fracture transversal to the film. d) Boxplots of resistance variation over the 50 measurement cycles for relative comparison (The samples with 47 Vol.% (n‐BiTeSe) is not represented due to the complete fracture of the film, reaching the overload of the equipment). Note: Boxplots have defined the minimum and maximum values marked with a horizontal line, and mean values marked with ★.

From an application point‐of‐view, textural properties are crucial. For this reason, flexibility analysis of the printed films was conducted based on Miriam Alvarado et al.^[^ ^45^ ^]^ work, and the results are depicted in **Figure** **5**. For the measurements, all the samples were subjected to 50 tensile cycles with a maximum displacement (Stroke) of 1 mm, which corresponds to a radius of curvature of 5.6 mm.

Figure 5a illustrates the initial 16 cycles for the sample containing 32 Vol.% (n‐BiTeSe), while Figure 5b displays specific points — maximum deformation, minimum deformation, and minimum resistance —across 50 cycles for the same sample. The first figure demonstrates a consistent rise in film resistance with applied deformation, followed by a gradual increase after each cycle. This escalation in resistance results from TE microparticle separation, intensifying contact resistance and generating irreversible microcracks within the films. In rigid samples, such deformation leads to visible cracks, depicted in Figure 5c. These cracks predominantly stem from the higher concentration of p‐BiSbTe material, rendering the film exceptionally fragile and yielding over a tenfold increase in resistance.

With the exception of the samples containing the highest concentrations of inorganic powder (38 and 47 Vol.%), all the remaining samples reached a flexibility regime after ≈40 cycles and maintained a stable resistance cycle after cycle. For example, in Figure 5b, the stabilization of the film resistance from 1000 s can be seen, indicating that the film acquired flexibility.

Figure 5d displays the Δ*R* results achieved for all the samples produced in this work, with the minimum, maximum and mean values marked. The results obtained illustrate the flexibility of the samples produced, with resistive variations remaining below two times for the majority of the samples. These variations are attributed to the micro‐cracks produced in the films, which do not prevent contact between the ends, confirming that the detection of temperature gradients remains unaffected by the bending of the films in the measured curvature range. Taking the analysis of the transport properties (Figure 4) and flexibility (Figure 5) into account, the films to be used in the final prototype will be 32 Vol.% (n‐BiTeSe) and 32 Vol.% (p‐BiSbTe).

## 3 Concept Validation for the Thermoelectric e‐Tattoo

### 3.1 Numerical Simulation

**Figure 6:**
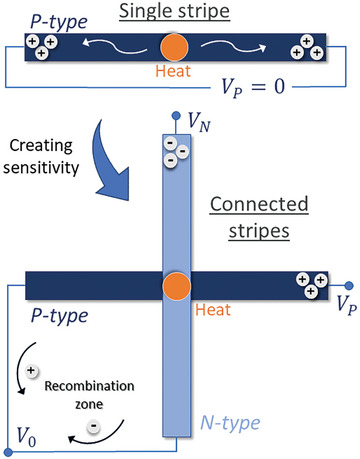
Proposed solution for creating sensitive points along the TE stripes. In a single stripe, it is not possible to obtain sensitivity in the center point. Using connected stripes with a 1s × 1s configuration, new points of sensitivity can be introduced.

The COMSOL Multiphysics platform, utilizing Finite Element Methods and modules like Energy Transfer and Thermoelectric, served as the tool for simulating and demonstrating the proposed proof‐of‐concept depicted in Figure 1 by using a 1s × 1s configuration (see **Figure** **6**). As observed in this figure, the applied heat in the center of the stripe will induce the movement of the charges for the two ends of the stripe in a similar way, not alloying the detection. Using connected stripes, it is possible to always maintain a reference at zero potential in the “recombination zone,” allowing the detection of temperature variations in the center and introducing a new point of sensitivity.

The TE stripes featured a thickness of 40 µm, a width of 3 mm, and a length of 50 mm. Bi_2_Te_3_, with *S* values of 150 and −150 µV K^−1^ for the p‐type and n‐type stripes, respectively, was selected for the investigation. A time‐dependent analysis aimed to identify and quantify the temperature change point. Standardized experiments involved a temperature alteration of Δ*T* = 6 K at 5 s and −6 K at 15 s. Electrical contacts, as depicted in Figure 6, were utilized to monitor the generated potential throughout the numerical simulation. An initial study was done to analyze the influence of the substrate, using Kapton and air (without substrate), concluding that both results were equal. In this way, to reduce the computing time, the absence of a substrate was chosen. Further details of the study can be found in Table S‐B1 in the Supporting Information.

**Figure 7:**
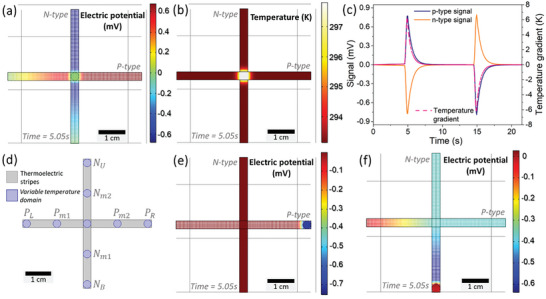
a) Electric potential distribution and b) temperature gradient along the stripes at 5.05 s of study for temperature change at the point of intersection of the stripes. c) Electrical signals obtained throughout the study at the central point by placing probes at the ends of the stripes. d) Nine different positions of the 1s × 1s configuration under study. e) Electric potential distribution for the 5.05 s of study causing temperature change in the right extreme, ***P***_***R***_, and f) in the bottom extreme, ***N***_***B***_.

In **Figure** **7**, the numerical outcomes pertaining to the temperature variation at the configuration's center are presented. At the 5.05 second mark of the study, images illustrating the potential gradient (Figure 7a) and *ΔT* (Figure 7b) were extracted. Furthermore, Figure 7c showcases the electrical signals acquired throughout the complete duration of the study.

The potential distribution image confirms that heating at the center of the configuration leads to the accumulation of negative charges at the upper end and positive charges at the far right side. However, at the other two extremes with the connection, the electrical potential remains at zero. Thus, signal analysis reveals the generation of a positive signal for the p‐type stripe and a symmetrical signal for the other stripe during the heating point. By calibrating the thermal sensitivity, *TS*, at this point, we obtained symmetrical values with an amplitude of 126 µV K^−1^, which is slightly lower than the *S* of the materials used. In addition to studying materials of different types, we also used materials of the same type, namely p‐type/p‐type and n‐type/n‐type. It was observed that the temperature change point led to a potential change, but the ends maintained the same electrical potential, making the *ΔT* impossible to detect. These findings support the concept described in Figure 6, emphasizing the importance of using the combination of n‐ and p‐type materials to detect gradients along the stripe.

In addition to the central point, other points of the configuration were also analyzed (see Figure 7d), obtaining sensitivity in 5 different points. For the upper extreme, denoted as *N_U_*, and the right side, denoted as *P_R_*, the phenomenon closely resembles the traditional Seebeck effect, as can be seen in Figure 7e,f. In the last case, the point with the highest temperature will have a deficit of free charges, leading to a change in electric potential, while the rest of the configuration will remain at zero potential. In such cases, only the temperature‐changed stripe will have an associated potential difference, allowing differentiation from the central case, in which both stripes have electrical signals with symmetrical amplitudes. Considering the proximity of the probe and point of temperature change, its sensitivity is approximately equal to *S*, and with the expected signal given the nature of the materials.

However, the scenario differs slightly for the lower extreme, *N_B_*, as shown in Figure 7f, and the left side, *P_L_*. In this case, charge diffusion occurs between the recombination zone, *V*_0_, and the central zone, resulting in a decrease in the measured signal in *V_N_* and *V_P_*, i.e., in sensitivity. Furthermore, the central connection induces the stripe to remain at the same temperature along its entire length, to mimic the stripe's behavior with temperature changes, resulting in equal signals for both stripes. The thermal sensitivities for the p‐type, *TS_P_*, and n‐type stripe, *TS_N_*, at different points were calculated using the obtained data, and the results are shown in the left side of Figure S‐B1 (Supporting Information). This image confirms that the temperature change point can be easily located and quantified using the relationship and direction of the signals obtained in the two stripes, confirming that it is possible to analyze 5 points using only 2 signals.

**Figure 8:**
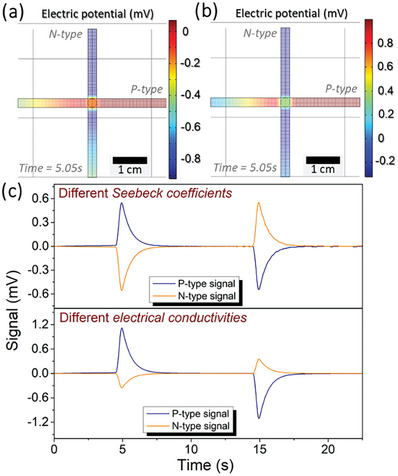
Numerical results obtained using materials with different properties. Electric potential distribution for the 5.05 s of study using materials with a) different Seebeck coefficients and b) different electrical conductivities. c) Electrical signals obtained throughout the entire study by placing probes at the ends of the stripes for the two cases analyzed.

In addition to using materials with the same properties, and considering that the materials to be used experimentally do not have the same characteristics, a study was carried out with materials of different *S*, placing S_N_ =  −170 µV K^−1^ and S_P_ = 50 µV K^−1^, and another study with different σ, with σ_N_ = 3σ_P_ . These values were chosen in order to simulate extreme situations with a high difference between materials. The results obtained are shown in **Figure** **8**.

In the context of materials exhibiting different coefficient values (*S*‐values), the material possessing the highest *S* coefficient will inherently produce a greater charge asymmetry. In this specific scenario, the n‐type material emerges as the one with the highest coefficient. Consequently, owing to this heightened charge asymmetry, there will exist a notably augmented density of free electrons within the recombination zone in comparison to the presence of holes from the p‐type material. This charge density difference will result in an excess of electrons in this zone after charge recombination, inducing *V*_0_ to be negative, as shown in Figure 8a. With the decrease of *V*_0_, the ΔV in relation to the other end of the n‐type stripe decreases, while it increases in relation to the other end of the p‐type stripe. This decrease occurs until it reaches the equilibrium point where these two potential differences become equal, that is, when *V*_0_ reaches the midpoint between *V_P_* and *V_N_*. Quantitatively, the magnitude of the *TS* with these characteristics is exactly the same for both stripes and equal to 87 µV K^−1^ (slightly lower than the midpoint of the Seebeck coefficients used), with the signals being completely symmetrical, as shown in Figure 8c.

A unique situation arises when there is a variation in σ among the materials. First, an equal charge imbalance is seen in both materials. More conductivity, on the other hand, indicates that there is a larger flow of charges (*n*) in the material (*σ* = n q µ). An imbalance, characterized by a surplus of electrons over holes in the recombination zone, can result under these conditions. This excess traditionally played a key role in balancing the threshold sensitivities across different stripes. Even so, in this case of constant charge asymmetry, electron buildup causes *V*_0_ to move from *V*_P_ to *V*_N_, disrupting the balance between sensitivity. Through the obtained signals, the *TS* obtained for the *n‐type* stripe is − 57.3 µV K^−1^, while for the p‐type stripe it is 180 µV K^−1^. That is, the most conductive material is the material with the lowest sensitivity, following the relationship in Equation 1.

(1) $$\frac{\sigma_{N}}{\sigma_{P}}\approx \frac{TS_{P}}{TS_{N}}$$

Considering that in the materials used experimentally, the greatest difference in properties is in the σ, the *TS* for the remaining points using different σ were also analyzed, and the results obtained are represented on the right side of Figure S‐B1 (Supporting Information). These results show, in addition to the *TS* asymmetry at the central point, a decrease in *P*_L_ sensitivity and an increase in *N*_B_ sensitivity when compared to the results obtained with materials with similar properties. These results are important because they indicate that new material combinations can be explored in future work to further optimize this original structure. For example, materials such as AgSe‐based compounds with higher *S*, ^[^ ^46^ ^]^ or low‐cost alternatives like PEDOT:PSS^[^ ^47^ ^]^ could be considered. The only limitations observed in this work are that the two materials must have opposite charge carriers (n‐type and p‐type material), and the ratio between the electrical conductivities cannot be too high.

### 3.2 Experimental Validation

For the validation of our concept and in alignment with the materials optimization phase, the selected films exhibiting the most promising combination of TE and mechanical properties are those comprised of 32 Vol.% (n‐BiTeSe) and 32 Vol.% (p‐BiSbTe). As a result, these films were used to produce the 1s × 1s configuration, as depicted in Figure S‐B2a presented in the supporting information document, featuring the same dimensions as the one used in the numerical simulation. Figure S‐B2b (Supporting Information) displays an optical microscope image of the intersection of the stripes, with the p‐type stripe on top of the n‐type stripe. To assess the sensor flexibility, this device can be bent in different ways, as shown in Figure S‐B2c (Supporting Information).

**Figure 9:**
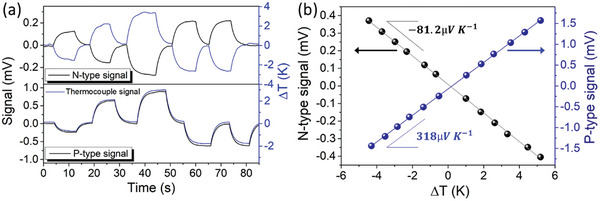
a) Thermoelectric signals obtained at the center of the sensor and comparison with signals obtained through the placement of thermocouples. b) Linearization of the data obtained to determine the sensitivity of each stripe.

To validate the sensitivity at the five numerically determined points, the Peltier module was placed under these same positions. Different Δ*T* values were applied to each point to obtain the sensor's sensitivity via data linearization. The results obtained for the central point of the sensor are represented in **Figure** **9**. In addition to the potential generated in each stripe, Figure 9a also illustrated the temperature variation curves using thermocouples. The comparison of sensor response to applied temperature gradients reveals an exceptional performance, showcasing response and reset times to those of commercial thermocouples. Additionally, the demonstrated stability of the TE sensor signal surpasses that of the thermocouple signal. These findings indicate that, in practical applications, the sensor's susceptibility to external thermal fluctuations is notably reduced. This characteristic enables precise monitoring of skin temperature, ensuring a higher degree of accuracy in various monitoring contexts.

The p‐type stripe signal exhibits a positive response in accordance with the numerical simulations, while the n‐type stripe signal shows a negative response. The linearization of the obtained values, presented in Figure 9b, confirms that *TS*_P_ = (318 ± 1) µV K^−1^ and *TS*_N_ = (81.2 ± 0.5) µV K^−1^. This asymmetry in sensitivities is attributed to differences in the electrical conductivities of the materials used, which have a ratio of ≈3, and as expected, the most conductive material has the lowest sensitivity. Although it is not possible to accurately determine the response time of the sensor from this figure due to the gradual temperature variation, a comparison with commercial thermocouples indicates that both performances are very similar for this type of slow variation. Considering the final application of this sensor, our sensor has ability to respond to the slow temperature oscillations of the skin.

In addition to the central point, the remaining points were also examined, and the results are presented in the left side of Figure S‐B3 (Supporting Information). This image shows that each point has a unique relationship between sensitivities, enabling clear differentiation. At the P_L_ point, it is confirmed that both stripes exhibit a positive sensitivity, albeit with a reduced amplitude, which compromises precise sensitivity. This decrease aligns with expectations, considering the difference in electrical conductivities between the materials. Conversely, at N_B_, both stripes demonstrate a negative response. However, at points P_R_ and N_U_, only one of the stripes responds to temperature changes. When comparing these observed values to the expected values at the ends of the stripes (right side of Figure S‐B1, Supporting Information), it becomes evident that the obtained values are lower than anticipated. This decline in sensitivity could be attributed to heat dissipation in the metallic contacts, resulting in a temperature difference between the underside of the substrate and the TE stripes where the temperature change occurs.

**Figure 10:**
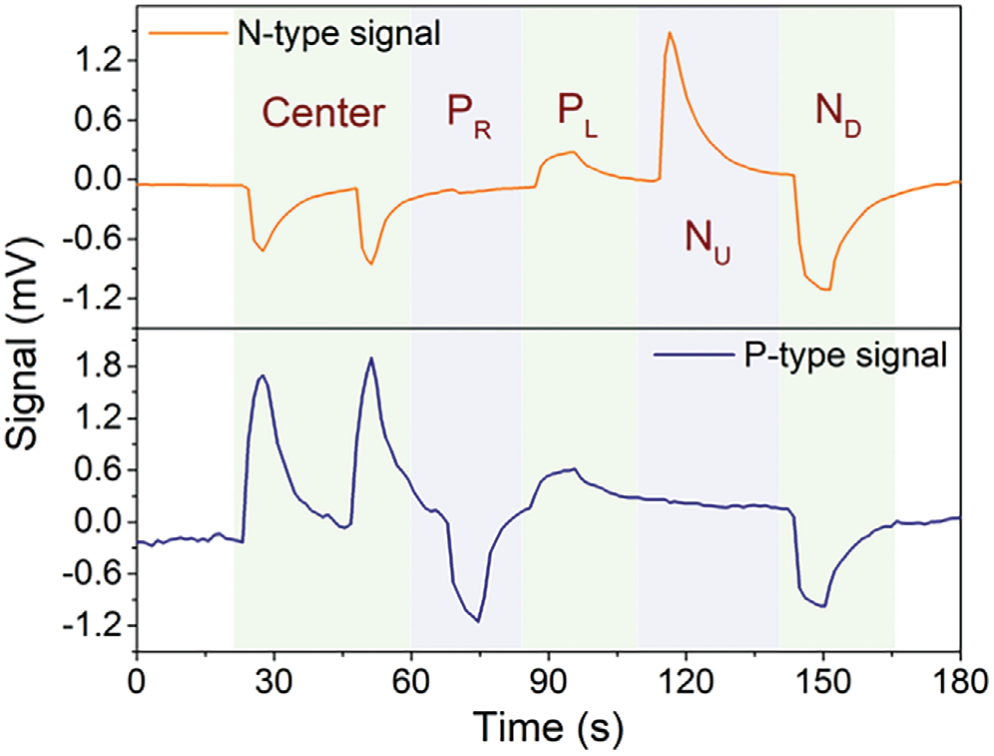
Final sensor test using a human finger to promote temperature changes at various sensor positions for comparison.

Following the validation of the sensor's functionality and the uniqueness of each point, a human finger was used to perform a sequential test on the sensor. This test is depicted in **Figure** **10**, and as can be observed, two touches were performed in the center of the sensor, followed by touching in the position *P*_R_, *P*_L_, *N*_U_, and *N*_D_, creating symmetrical responses in both stripes, a response only on the p‐type stripe, positive response in both stripes, response only in the n‐type stripe, and a negative response in both stripes, respectively. In terms of response time, the time differences between the application of temperature and the point at which the signals achieved 90% of their final response was between 2.5 and 3 s, indicating that the time response of the sensor to instantaneous variation of temperature is lower than 3 seconds.

Based on the results presented, it is possible to conclude that the developed sensor exhibits sensitivity in 5 different points, as well as excellent linearity and response time. Furthermore, it is noteworthy that the numerical findings presented have been experimentally validated.

## 4 Prototyping: Temporary e‐Tattoo and Adaptable Dressing

After conducting a fundamental investigation into the 1s × 1s, the first prototype was printed onto tattoo paper for temporary skin application to validate the functionality of the developed sensor. Figure 13a delineates the process used to fabricate the e‐tattoo sensor. The procedure entails the following steps: i) screen‐printing TE stripes and contacts onto the white segment of the tattoo paper, ii) establishing electrical connections using 50 µm Cu wire, employing Ag ink for contacts, and securing them with Kapton tape to prevent displacement, iii) applying an adhesive layer that remains positioned between the skin and the sensor, and iv) situating the tattoo paper on the skin and moistening it to aid in removing the white section of the tattoo paper. Figure 13a‐v portrays the final sensor, printed on the inner forearm, shielded by two thin layers, ensuring no direct contact with human skin or the external environment.

Sensor calibration was carried out for all positions using the inks and substrate as the sensor implemented on the skin. However, in this instance, the Peltier was placed directly onto the TE stripes, without any intermediate layer (such as Kapton), and without the use of planar metallic contacts, as was previously employed. The calibration results are represented on the right side of Figure S‐B3 (Supporting Information), reveal that the relation between the sensitivities of each stripe aligns with the expected values for different points. The high magnitude of the sensitivities is related to the direct contact between the Peltier face and the TE material and the lack of metallic contacts, which prevents thermal dissipation of heat.

**Figure 11:**
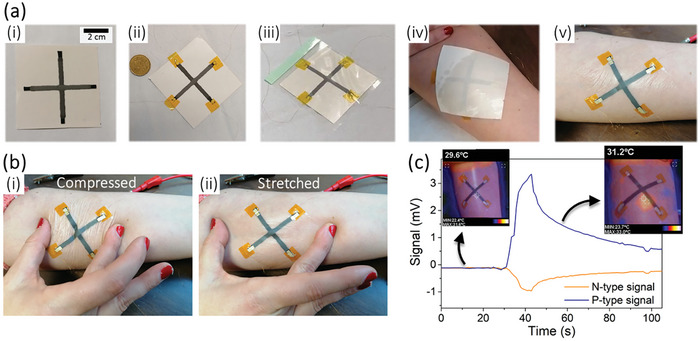
a) Production of the sensor and transfer to the skin: i) screen‐printing of the thermoelectric stripes and contacts, ii) placement of the contacts using 50 µm Cu wire, Ag ink for contacts and Kapton tape, iii) placement of the layer between the sensor and the skin, iv) placement of the tattoo paper in the skin and humidification, v) printed sensor obtained on the surface of the skin. b) Mechanical deformations applied to the sensor to confirm stability: i) Compressed, ii) Stretched. c) Thermoelectric signal obtained with the application of a temperature gradient in the center of the configuration and respective thermal images.

Following calibration and to assess the mechanical stability of the sensor integrated into the skin, both mechanical compression and stretching deformations were performed, as represented in **Figure** **11**(b‐i) e (b‐ii), respectively. Simultaneously, the resistances of the stripes were monitored. No visible cracks were observed in the TE stripes, and in quantitative terms, the resistances increased between 4% and 5% at the moment of the deformation, remaining approximately the same after the movement (see Figure S‐B5a, Supporting Information).

The Peltier module, which was previously calibrated, along with a thermal camera, was used to analyze the sensor's response to temperature gradients on the skin's surface. Initially, the temperature of the skin's surface was measured and confirmed to be ≈30 °C, with a uniform temperature across the surface. The Peltier module was placed at 41 °C on the hot surface, and this surface was then placed against the center of the sensor until it reached a regime of quasi‐stabilization, which took ≈10 seconds. After heating, the skin was allowed to cool naturally without the use of any external mechanisms. Figure 11c depicts the signal obtained in this test, along with two thermal images. These images were captured both before heating with the Peltier module, when the skin had a uniform temperature, and after heating, while the skin was cooling down. The slight phase difference between the thermal image and the regular image can be attributed to the different positions of the visible and infrared cameras. Nevertheless, it is worth noting that the hottest spot on the sensor corresponded to the intersection of the stripes. The maximum values of the different signals were *V*_p_ = 3.34 mV and *V*_n_ = 0.96 mV, which was expected given the applied Δ*T* and the sensitivities of the central point (as shown in Figure SB3, Supporting Information). This test confirms that the sensor responds to temperature gradients when applied to the skin's surface.

Furthermore, the sensor's ability to detect temperature gradients may be indicative of potential inflammatory conditions, suggesting its utility in identifying such physiological changes. The examination of its response to a brief touch with a finger that was slightly warmer than the skin's surface (between 1 and 2 °C warmer) was conducted as well. The results obtained are depicted in Figure S‐B4 (Supporting Information). The signals obtained can be used to confirm the existence of peaks whenever a touch is made at the intersection of the stripes, although with more noise than previously due to the lower *ΔT* used.

**Figure 12:**
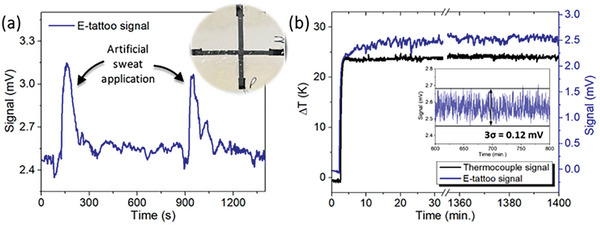
a) Variation of the p‐type signal with the application of commercial artificial sweat to the center of the e‐tattoo, with a constant temperature gradient applied across it. b) Determination of the minimum detectable temperature fluctuation by the e‐tattoo using the standard deviation (σ) of the noise under a stable temperature gradient.

In summary, the conducted tests validate the efficacy of the proposed configuration, demonstrating the feasibility of analyzing 5 points on the skin's surface using only two TE signals. Through the interpretation of the generated signals, it becomes possible to reconstruct the temperature gradients and distinguish touch and infections through the duration and shape of the signals. To finalize the proof‐of‐concept of this approach, additional tests were conducted in terms of mechanical stability, resistance to sweat, and minimum temperature fluctuation detectable by the produced e‐tattoo. The results are presented in **Figure** **12** and Figure S‐B5 (Supporting Information). As previously mentioned, the films are incorporated between two thin layers. These layers are part of the commercial tattoo paper, providing impermeability and ensuring extra stability and protection against external environmental factors. This was confirmed in the mechanical tests represented in Figure S‐B5b (Supporting Information). This figure illustrates the resistance variation of each individual TE stripe printed on the tattoo paper with the same contacts as the final e‐tattoo and with the two protection layers above and below the films. The samples were bent until a curvature radius of 5.6 mm, as previously mentioned, and compared with the results from Figure 5d for the same material concentration. These samples present higher stability. On the tattoo paper, the resistance increase is up to 2 times the initial value, while in Kapton, it rises up to 7 times the initial value. The resistance variation is of the same order of magnitude for both the p‐type and n‐type stripes, so it is not expected to influence the sensor performance.

The resistance to sweat was analyzed by spraying artificial sweat directly onto the tattoo layer that contacts the skin accordingly *DIN EN 1811:2023‐04*. Initially, the stripes were analyzed individually, and their resistance was monitored over time following the application of sweat. The results are represented in Figure S‐B5c and d (Supporting Information). Upon spraying the sweat, an increase in resistance was observed, proportional to the quantity of droplets on the surface. However, after some time, the sweat evaporates, and the resistance returns approximately to its initial value. Similar to the mechanical deformation, the resistance variation in this case is consistent for both materials (less than 9 times the initial value) and thus is not expected to influence the sensor signals. To confirm that the signals remain unaffected by the amount of sweat, sensors printed on tattoo paper and confined within the two thin layers were exposed to sweat, with a *ΔT* applied across the same. Figure 12a shows the variation of the p‐type stripe signal over time. After the *ΔT* and signal stabilized, artificial sweat was applied twice to the center of the e‐tattoo. Then, a voltage increase was observed, however, in comparison with the relaxation time shown in Figure S‐B5c and d (Supporting Information) (up to 20 min), in this case, the signal recovered much faster (up to 200 s). This indicates that the oscillation is due to the slight temperature difference between the sweat and tattoo surface rather than resistance variations. After this sweat test, the e‐tattoo maintained its performance.

To complete the e‐tattoo characterization, the minimum temperature fluctuation detectable by the sensor was estimated. For this, the e‐tattoo was exposed to a constant temperature difference for 24 h in a room with natural temperature fluctuations. The minimum detectable temperature fluctuation was then calculated from the standard deviation (σ) of the noise. This noise includes contributions from the measurement equipment and the ambient temperature variations around the e‐tattoo. The results for the first 30 minutes and the last 40 minutes are represented in Figure 12b. The obtained 3σ value was 0.12 mV, corresponding to a minimum detectable temperature fluctuation of 0.4 K.

**Figure 13:**
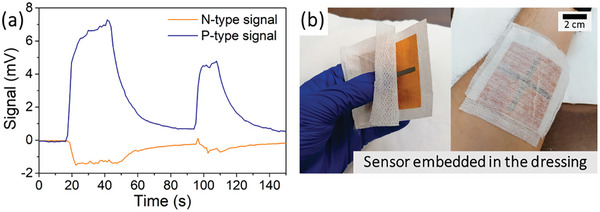
Adaptation of a produced sensor for use inside a dressing, with signal generation via temperature application in the dressing's center: a) generated signals, and b) device application.

Depending on the application, especially in chronic wound healing, directly printing of these sensors onto the skin is not medically recommended. Thus, these flexible sensors can be adapted for use in alternative scenarios, such as inside dressings, as shown in Figure 13b. In this scenario, there is a reduction in sensitivity due to reduced skin contact, which depends on the thickness of the underlying cotton layer. Nevertheless, even with this layer, it remains possible to detect heat, as illustrated in **Figure** **13**a. These peaks are the result of applying heat with a Peltier module to the dressing containing the sensor.

Compared to the sensors reported in the literature and summarized in Table 1, the configuration proposed in this work offers several notable advantages. As discussed earlier, the TE signals generated are independent of resistive variations in the materials, making them immune to interference from movement or the presence of sweat on the surface of the e‐tattoo. This characteristic provides a substantial improvement over traditional resistive sensors. Furthermore, the proposed configuration enables the analysis of horizontal temperature gradients across the skin, with the capacity to analyze more data points than twice the number of signals with just two stripes. The number of data points increases quadratically with the number of intersections. For instance, by utilizing three p‐type and three n‐type stripes connected at one end, as depicted in Figure 6, it is possible to monitor 21 distinct points simultaneously using only six signals (refer to Figure SB6, Supporting Information). This capability is highly advantageous for monitoring large surface areas while minimizing the volume of data that needs to be processed. Thus, the configuration presented here represents a significant advancement over previously reported TE sensors in the literature.

## 5 Conclusions

The presented work encompasses a series of pivotal processes aimed at developing a flexible and transient thermal sensor. These processes involved the careful optimization of material properties, the use of numerical simulations to evaluate the proposed configuration, and the prototyping phase to establish proof‐of‐concept. Our research used p‐type Bi_0.35_Sb_1.65_Te_3_ and n‐type Bi_2_Te_2.8_Se_0.2_ powder, combined with PVA doped with H_3_PO_4_, for screen‐printing. We found that while S remained constant with changes in TE powder concentration, the σ varied depending on print quality and material concentration. After analyzing both flexibility and degradation over time, we determined that an optimal concentration of 32 Vol.% for p‐BiSbTe and n‐BiTeSe provided excellent TE performance and mechanical durability.

After material optimization, we conducted numerical simulations under conditions similar to the real material characteristics. The results confirmed that the use of transverse stripes led to an increase in the ratio of sensitivity points to signals, reducing the need for multiple electrical contacts. This advantage distinguishes our approach from those found in the literature. The 1s × 1s configuration presented 5 sensitivity points, each easily distinguishable. Additionally, when using materials with different transport properties, it was observed that the signals maintained symmetrical sensitivities for different S, while σ causes an asymmetry in the sensor's sensitivities.

To validate the numerical results obtained, we developed experimental prototypes as a proof‐of‐concept. The first prototype, produced on Kapton, confirmed the compatibility with the numerical results. After calibration, the sensitivities for the central point were found to be asymmetric due to the difference in the conductivities of the materials, with sensitivities *TS*_P_ = 318 µV K^−1^ and *TS*_N_ =  −81.2 µV K^−1^. The second sensor, transferred to the skin using temporary tattoo paper, was tested for its response to a simulated infection on the skin's surface. Sensitivities for the center point were *TS*_P_ =  279 µV K^−1^and *TS*_N_ =  −80.3 µV K^−1^, with a maximum sensitivity of 230 µV K^−1^ and a minimum of 24.5 µV K^−1^ at the edges. The minimum detectable temperature fluctuation was 0.4 K, and the sensor resistance to artificial sweat was confirmed. The sensor also showed a fast response time of less than 3 s when subjected to a finger touch. These results validate the practicability of the proposed configurations, confirming their responsiveness to both temperature changes and external stimuli. The linear relationship between resistance variation and applied deformation in the flexibility tests further suggests the adaptability of these materials to dual‐function sensors capable of detecting both temperature and movement. Moreover, considering the use of TE materials, the sensor can be adapted as a self‐powered device, enhancing user comfort in temporary e‐tattoo applications. This work opens new possibilities for the development of TE planar e‐tattoos in the future.

## 6 Experimental Section

### Thermoelectric Powder

The n‐type and p‐type Bi_2_Te_3_ powder were purchased from the company “Wuhan Xinrong New Materials Co. Ltd.,” with a purity of 99.99% and a particle size of 325 mesh (<44 µm). These Bi_2_Te_3_ powders were pressed into pellets for further characterization. A press‐machine was employed to apply 4000 psi for 30 s at room temperature to compact the TE powders into pellets. The resulting pellets had a diameter of 1.3 cm, and their thickness, denoted as *t*, was determined using a numerical indicator with an accuracy of 1 µm.

### Formulation of the Doped‐Polymer

For the ink production, liquid PVA doped with H_3_PO_4_ was used. The solid PVA with 99% purity and the liquid H_3_PO_4_ with a concentration of 85% were acquired from Sigma–Aldrich and Fisher Chemical, respectively. To prepare the polymer solution, the solid PVA was mixed with the H_3_PO_4_ and ultra‐pure water − Millipore water − with a mass ratio of 1:1:1. The resulting mixture was then heated up to 85 °C in a silicone bath and left to dissolve and homogenize for 6 hours. Once fully dissolved, the polymer solution was left to cool down to room temperature.^[^ ^37^ ^]^

### Manufacturing of Printed Films

To produce the TE ink, different mixtures of Bi_2_Te_3_ and PVA/H_3_PO_4_ were prepared, specifically with 19, 22, 27, 32, 38, and 47 Vol.% of inorganic powder, corresponding to 60, 65, 70, 75, 80, and 85 wt.%. These obtained inks were printed, after being well mixed, on precleaned Kapton substrate with a thickness of 50 µm, using the screen‐printing technique with a mesh‐opening of 249 µm − PET 1500 27/120 W mesh. The resulting samples had a width of 1 cm, a length of 2 cm and variable thickness, which was measured using a numerical indicator. After printing, the films were dried at room temperature. More information regarding the printing process could be found in Figure S‐B7 (Supporting Information).

### Characterization of the Thermoelectric Samples

SEM was employed to analyze the surface morphology of the samples. The p‐type and n‐type samples were analyzed using the Scios 2 Hi Vac de Thermofisher Scientific with an Energy‐Dispersive X‐ray (EDX) detector from Thermofisher at the Universitat Rovira i Virgili (URV) facilities, and the HITACHI FlexSEM 1000 with Bruker EDX detector at the Department of Geosciences, Environment and Spatial Plannings, Faculty of Science, Porto University, respectively.

For the crystalline characterization, the D8‐Discovery diffractometer equipment with the detector LynxEye‐XE‐T was used from the p‐type samples at the URV facilities, and the high‐resolution SmartLab Rigaku diffractometer from the Institute of Physics for Advanced Materials, Nanotechnology and Photonics (IFIMUP) facilities was used for the n‐type samples. Both equipment's employ the Bragg‐Brentano θ − θ configuration and the Cu K‐α radiation ($\lambda =1.540593\;\dot{A}$), with a Cu X‐ray tube operating at 40 kV.

The transport properties, namely *S* and σ were evaluated in both produced samples, namely the pellets and the printed films. Regarding the measurement of *S*, a home‐made set‐up consisting of two independent and isolated Cu pillars was employed. The independent samples were placed on top of the pillars and supported at their ends. A Δ*T* was applied to the ends of the samples using a heater and a thermocouple (Type K) connected to the temperature controller (Lake Shore 331 TC). The generated Δ*V* in the sample was measured using a nanovoltmeter (Keithley 2182). Both devices were controlled by a LabVIEW routine, which applied several temperature gradients to obtain *S* through data linearization (*S* = −Δ*V*/Δ*T*).^[^ ^44^ ^]^

The 4‐contact method was employed to analyze the electrical resistivity, ρ, of the samples. The current source used was the DC Current and Voltage Calibrator from Time Electronics, and the potential generated was measured with a Keithley 2182 nanovoltmeter. To determine ρ, the correction factors, *F* = *F*_1_ *F*_2_ *F*_3_ defined for non‐infinite samples were used.^[^ ^48^ ^]^ In this work, *F*_3_ can be considered equal to 1. However, *F*_1_ and *F*_2_ are determined by the following Equations 2 and 3, respectively:

(2) $$F_{1}=\frac{t/s}{2\ln\left(\frac{\sinh\left(t/s\right)}{\sinh\left(t/2s\right)}\right)}$$

(3) $$F_{2}=\frac{\ln\left(2\right)}{\ln\left(2\right)+\ln\left(\frac{{\left(D/s\right)}^{2}+3}{{\left(D/s\right)}^{2}-3}\right)}$$

In these equations, *t* corresponds to the thickness of the samples, *s* to the distance between probes and *D* to the diameter of pellets or the width of the rectangular films.

By using these correction factors, the resistivity can be determined by Equation 4, where *I* is the electrical current applied, and *V* the measured voltage drop.

(4) $$\rho =2\pi sF\frac{V}{I}$$

The *σ* is the inverse of ρ, represented as σ =  1/ρ. To measure the flexibility of the films, the AGS‐X 10 kN Shimadzu universal testing machine controlled by the Trapezium X Materials testing software was used for the mechanical deformation. The electrical resistance measurement was carried out using a Keysight 34972A multimeter.

### Thermoelectric Sensors

Manufacturing: For the proof‐of‐concept, a TE sensor was developed. To create the sensors, two substrates were used, namely Kapton and Silhouette tattoo paper. The optimized TE ink with 32 Vol.% of n‐BiTeSe and 32 Vol.% of p‐BiSbTe was used for the printing process. The screen printing procedure used a mesh‐opening of 249 µm (PET 1500 27/120 W). The fabrication started with the printing of an n‐type stripe with 3 mm of width and 50 mm of length. Subsequential, after the previous stripe dried at room temperature, a p‐type stripe with the same dimensions was printed transversely and superimposed on the central point of the first stripe. Following printing, the stripe was left to dry at room temperature.

In the case of the sensor printed on Silhouette tattoo paper, additional screen‐printing electrical contacts were incorporated to ensure stable connections after transferring the sensor to the skin. These contacts were designed with 3 mm of width and 5 mm of length, where 2 mm were superimposed on the TE stripes. These contacts were created using a layer of commercial Ag ink (VFP Ink Technologies) and another layer with carbon ink (Bectron GP 9553 VP) to enhance the interface stability between the Ag ink and the TE stripe. Cu wires were placed at the ends of the contacts and fixed using commercial conductive Ag ink obtained from the company “Agar Scientific” and covered with Kapton tape for insulation and stability, as illustrated in Figure 11.

### Characterization

To calibrate the developed sensor in Kapton, a 5 × 5 cm^2^ heatsink and a 1 × 1 cm^2^ Peltier module was placed underneath the sensor (see Figure S‐B8, Supporting Information). To improve the thermal contacts between the different elements of the setup, thermal paste was applied at the interface of the Peltier module with the heatsink and with the sensor. Additionally, a Type K thermocouple was installed for temperature control, while a second thermocouple was placed at the sensor's ends to analyze the generated ΔT. These thermocouples were connected to a PicoLog data logger. A DC Current and Voltage Calibrator from Time Electronics was used to power the Peltier module. To measure the ΔV generated along the sensor, a Keithley 2182 nanovoltmeter was used and controlled using a LabView routine. For measuring the e‐tattoo, the same equipment was used. However, to control the temperature applied with the finger, an infrared camera − Thermal Imaging Camera HT‐02D − was used.

The mechanical flexibility of the sensor stripes printed in tattoo paper was evaluated using the AGS‐X 10 kN Shimadzu universal testing machine with the Trapezium X Materials testing software and using the Keysight 34972A multimeter to measure the electrical resistance. The sweat resistance test was done by spraying a commercial synthetic sweat DIN 53 160:2023‐07 from Synthetic Urine while measuring the electrical resistance and voltage using the Keysight 34972A multimeter.

## Conflict of Interest

The authors declare no conflict of interest.

## Author Contributions

The manuscript was written through contributions of all authors. All authors have given approval to the definitive version of the manuscript.

## Supporting information



Supporting Information

## Data Availability

The data that support the findings of this study are available from the corresponding author upon reasonable request.
