# Supplementary material for: Touch Empowerment: Self‐Sustaining e‐Tattoo Thermoelectric System for Temperature Mapping
Source: Adv Sci (Weinh). 2024 Dec 26;12(7):2403775. doi: 10.1002/advs.202403775 (PMC11831475; doi:10.1002/advs.202403775)
Supplement: Supplementary file 1 — Supporting Information [file ADVS-12-2403775-s001.docx]

#

**SUPPORTING INFORMATION**

**Touch Empowerment: Self-Sustaining *e*-Tattoo Thermoelectric System for Temperature Mapping**

M. A. S. Almeida^1^, A. L. Pires^1*^, J. L. Ramirez^2^, S. B. Malik^2^, S. de la Flor^3^, E. Llobet^2^, A. T. Pereira^4^, and A. M. Pereira^1#^^[[1]](#footnote-1)^

^1^IFIMUP $-$ Physics for Advanced Materials, Nanotechnology and Photonics, Department of Physics and Astronomy, Faculty of Sciences, University of Porto, Rua do Campo Alegre, 4169-007 Porto, Portugal

^2^MINOS $-$ Universitat Rovira i Virgili, Avda. Països Catalans, 26, 43007 Tarragona, Spain

^3^Department of Mechanical Engineering - Universitat Rovira i Virgili, Avda. Països Catalans, 26, 43007 Tarragona, Spain

^4^i3S $-$ Institute for Research and Innovation in Health, Rua Alfredo Allen 208, 4200-135 Porto, Portugal

**Appendix A – Commercial Powder Characterization**

The two commercially acquired powders composed of Bi_2_Te_3_ micro-particles family, namely *p- type* and *n-type* powders, were characterized. These powders were denominated as *p*-BiSbTe and *n*-BiTeSe, respectively. The Figure S-A1-(a) and (b) display images obtained through Scanning Electronic Microscopy (SEM) of the two powders, which were used to analyze their size and morphology. The micrographs reveal that, in both cases, the particles exhibit significant variation in size and lack a well-defined morphology. The average particle diameter measures around of 44 µm with irregular geometries.


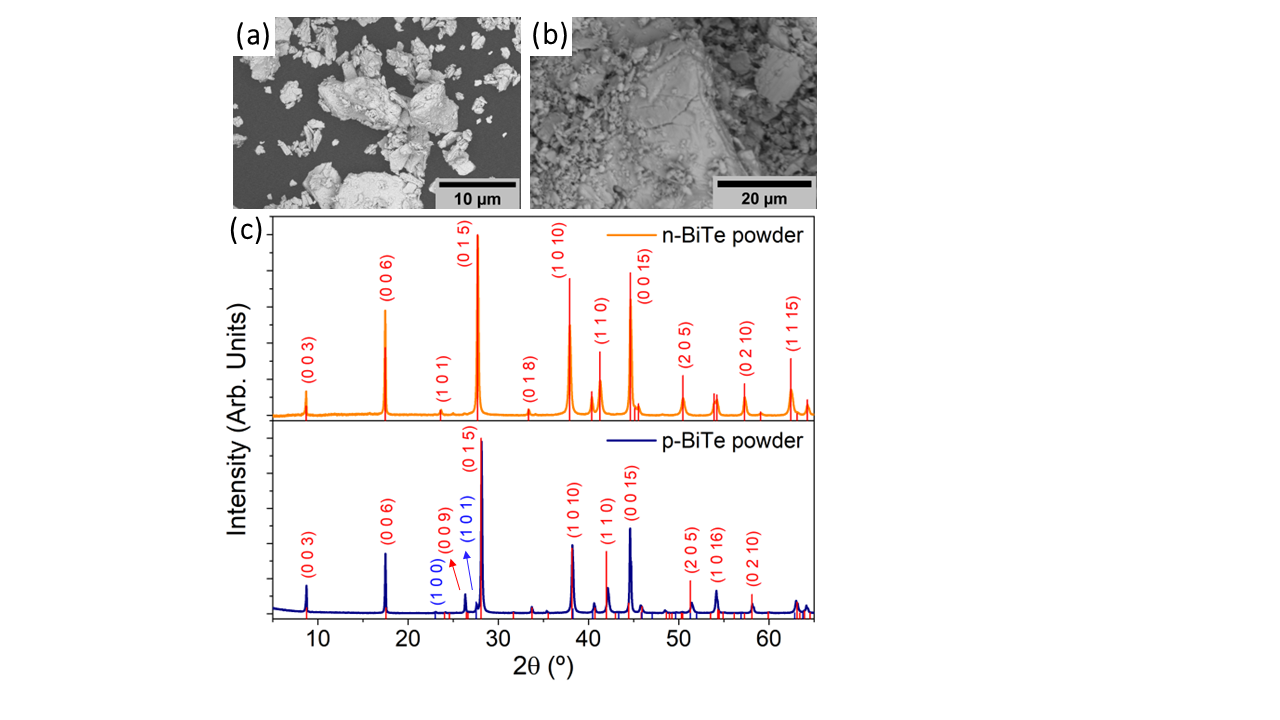


**Figure S-A1.** Characterization of commercial powders used in this work. (a) SEM image of p-BiSbTe powder with 5000x amplification; (b) SEM image of n-BiTeSe powder with 5000x amplification; (c) Diffractograms of the commercial powders with their respective Miller indices.

The structural characterization of the commercial powders was also performed through X-ray diffractogram (XRD) patterns, and the results are shown in Figure S-A1-(c). From this figure, it is possible to verify that both commercial powders exhibit a high degree of polycrystallinity and identify the present phases. Regarding for the *p*-BiSbTe powder, several Bragg reflections can be identified and indexed to two crystalline phases were identified. The majority of the peaks were determined to be from the crystalline phase of Bi_0.4_Sb_1.6_Te_3_ with a rhombohedral structure and space group $R\bar{3}m$ (identified by the vertical red lines in the graph). Additional reflections were also observed, which did not belong to the Bi_0.4_Sb_1.6_Te_3_ phase but to the Te phase with space *group* $P3_{1}21$ (identified by the vertical blue lines in the graph). For the *n-*BiTeSe powder, it was possible to identify several peaks reflections were identified to be coinciding indexed with the phase of the Bi_2_Te_3_ phase, featuring a rhombohedral $R\bar{3}m$ space-group structure. Both powders exhibit a preferred orientation along to the Miller indices (0 0 l), which can be attributed to the orientation during the preparation process due to their layered geometry.

Figure S-A2 shows the mapping of the elements presented in the thermoelectric samples acquired with Energy Dispersive Energy (EDS) analysis, specifically for *p*-BiSbTe and *n*-BiTeSe powders represented in Figure S-A2-(a) and Figure S-A2-(b), respectively. From these images, it is possible to detect the presence of the main elements of the Bi_2_Te_3_ family, which include Bi, Te, Se, and Sb. The uniform coloring of the images confirms the homogeneous distribution of these detected elements.


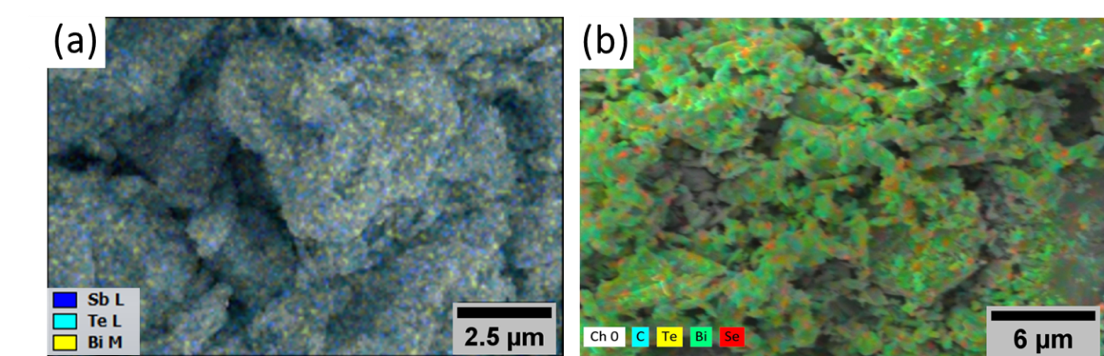


**Figure S-A2.** EDS mapping images of (a) p-BiSbTe and (b) n-BiTeSe powder, concerning the Bi, Se, Te, C, and O detected elements.

Table S-A1 summarizes the quantitative data from the EDS analysis, including the mass percentage (weight %) and atomic percentage (Atom %) of each detected element. The stoichiometry of the *p*-BiSbTe and *n*-BiTeSe powder samples was calculated by normalizing the Atom %. The presence of C and Al elements results from impurities introduced during the manufacturing process, and for those reasons, we exclude them from the calculations. For the *p*-BiSbTe powder, the data were analyzed considering the Bi_x_Sb_2-x_Te_3_ stoichiometry. It was observed that the powder is composed by Bi_0.35_Sb_1.65_Te_3_ along with an excess of Te, a conclusion confirmed also by XRD analysis. As for the *n-*BiTeSe powder, the results were adjusted to the Bi_2_Te_3- x_Se_x_ stoichiometry, yielding Bi_2_Te_2.8_Se_0.2_. Combining the information from the EDS and X-ray analysis, it was possible to confirm the *p*-BiSbTe powder is composed of Bi_0.35_Sb_1.65_Te_3_, and the *n*-BiTeSe powder of Bi_2_Te_2.8_Se_0.2_.

**Table S-A1.** Resumes the elements, Weight%, and Atom% achieved for the p-BiSbTe and n-BiTeSe powder through EDS analysis

| ***p*-BiSbTe powder** | | | |
| --- | --- | --- | --- |
| **Element** | **Weight %** | **Atom %** | **Norm. Atom %** |
| C | 1.465 | 13.91 | ------ |
| Al | 0.13 | 0.535 | ------ |
| Sb | 29.12 | 27.28 | 1.65 |
| Te | 58.77 | 52.535 | 3.18 |
| Bi | 10.52 | 5.74 | 0.35 |
| ***n*-BiTeSe powder** | | | |
| **Element** | **Weight %** | **Atom %** | **Norm. Atom %** |
| C | 0.88 | 10.94 | ------ |
| Se | 1.68 | 3.18 | 0.20 |
| Te | 42.7 | 50.0 | 2.80 |
| Bi | 50.17 | 35.87 | 2.00 |

**Appendix B – Numerical and experimental considerations**

Table S-B1 outline the numerical simulation parameters employed to obtain the numerical results presented in this work for the 1s×1s configurations.

**Table S-B1.** Parameters used for the numerical simulations

| **Software** | COMSOL Multiphysics 5.6 |
| --- | --- |
| **Module** | Thermoelectric effect with the respective physics interface (Heat Transfer in solids, Electric currents, and Electromagnetic Heating) |
| **Method** | Finite Element Method (FEM) |
| **TE equations solved** | $\left\{ \begin{aligned} \rho C_{p}\frac{\partial T}{\partial t}+\nabla\cdot\mathbf{q}=Q \\ \mathbf{J}=-\sigma\left( \nabla V+S\nabla T \right) \end{aligned} \right.$  Where $\rho$, $C_{p}$, $T$, $\mathbf{q}$**,** $Q$, $\mathbf{J}$**,** $\sigma$, $V$, $S$ is the mass density, heat capacity, temperature, conductive heat flux, Joule heat source, electric current density, electrical conductivity, potential difference and Seebeck coefficient, respectively. |
| **Study** | Time dependent study |
| **Computational mesh** | Physics-controlled with Finer mesh |
| **Convergence criteria** | Automatically provided by the program |
| **Materials properties** | Bi_2_Te_3_ properties imported from the materials library, changing the *S* to $150 \mu V K^{-1}$ and -$150 \mu V K^{-1}$ to the *p-*BiTe and *n-*BiTe material, respectively |
| **Boundary conditions** | The surface of the system was considered thermally isolated, with the assumption of the system in vacuum and initial temperature of $293.15 K$, with exception of the points with temperature variation. In these locals were considered a circular domain following one function with initial temperature of $293.15 K$, increasing this temperature by $\Delta T$ at 5 seconds and $-\Delta T$ at 15 seconds of study, followed by an exponential decrease unit the initial temperature. The $\Delta T$ values utilized was $6K$ to the analyse of each point’ response, and $[2 K, 4 K, 6 K]$ to confirm the response linearization. Regarding electric isolation, every system was considered isolated except for the lateral sides of the stripes’ ends. In the left ends and bottom ends, the lateral sides were considered as potential reference and electrical connected. In the right and top ends, the sides were considered as ‘*Floating potential*’. |
| **Outputs results** | To analyze the temperature gradient generated, was used temperature boundary probes in the ends of the stripes and in the domains with temperature variation. Regarding the potential difference generated, was used potential boundary probes in all the stripes’ ends and calculated the difference by the subtraction of the exported values. To have a spatial visualization of the temperature and potential in the configuration, was exported the $3D$ images of the temperature and electric potential with 100 X-planes, 100 Y-planes and 1 Z-Plane at 5 seconds of study. |

Figure S-B1 shows the numerical sensitivities obtained for five different points of the proposed configurations using materials with the same conductivities and symmetric Seebeck coefficients (left side), and with different conductivities and symmetric Seebeck coefficients (right side). For each point is indicated the sensitivity for the *p-type* and *n-type* signal.


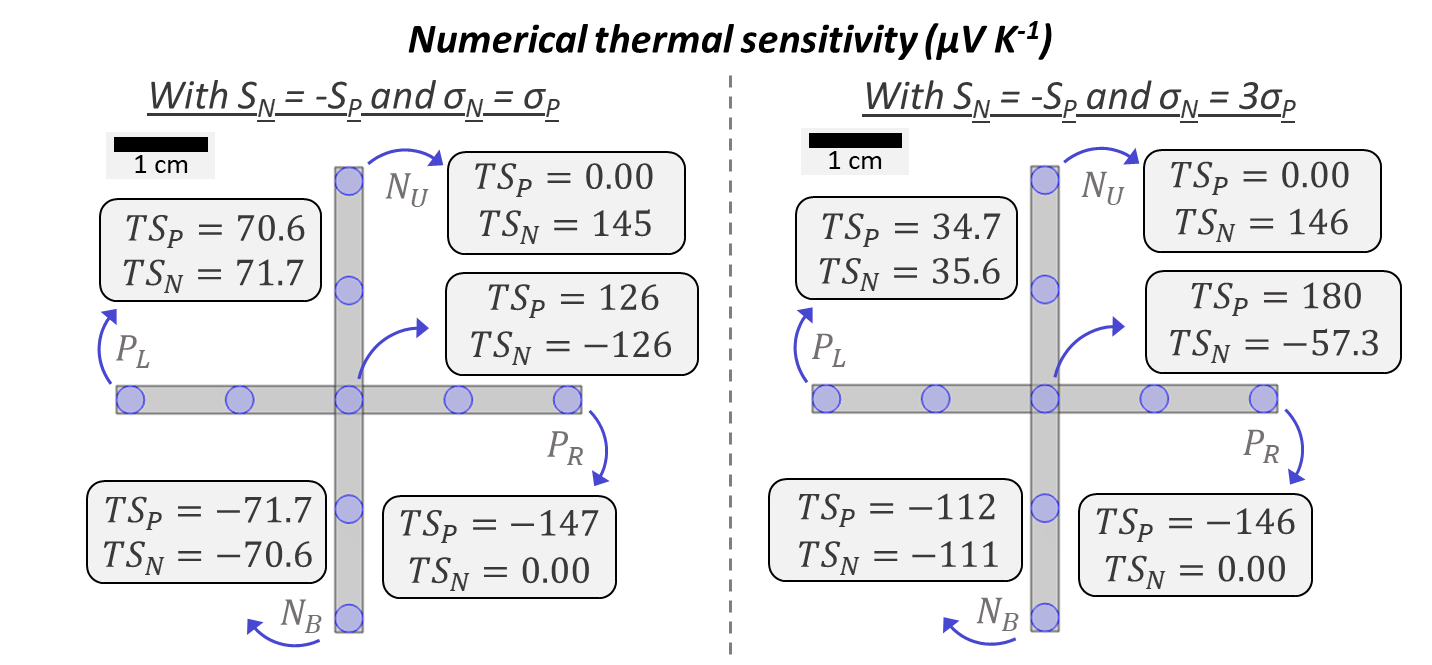


**Figure S-B1****.** Obtained sensitivities for the 5 sensitive points of the 1s×1s configuration in µV K^-1^. ($P_{L}$= left extreme; $P_{R}$= right extreme; $N_{B}$= bottom extreme; $N_{U}$= upper extreme; Center = intersection point of stripes).

Figure S-B2-(a) shows the device produced with the optimized TE inks on a Kapton substrate for validation with the same dimensions as the one used in the numerical simulations. Figure S-B2- (b) corresponds to an optical microscope image of the device center part, that is, to the intersection of the stripes. In Figure S-B2-(c), it is possible to confirm the device flexibility without any damage to the films.


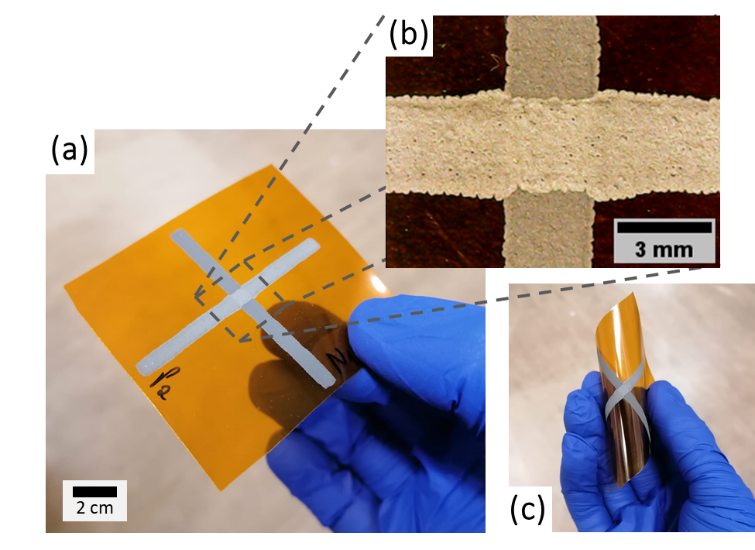


**Figure S-B2.** (a) Developed sensor printed in Kapton; (b) Image of the stripes central intersection using an optical microscope; (c) Bending of the sensor to confirm flexibility.

Figure S-B3 shows the experimental sensitivities obtained for the sensors produced in Kapton (left side) and tattoo paper (right side) in different points. For each point is indicated the sensitivity for the *p-type* and *n-type* signal.


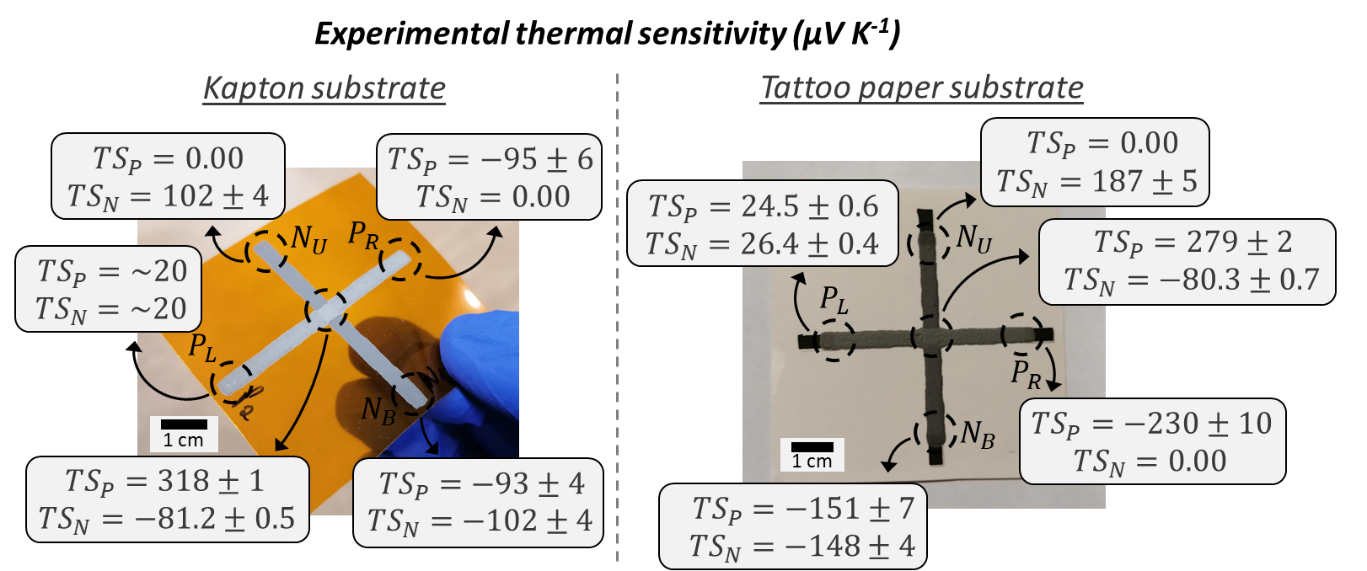


**Figure S-B3.** Sensitivities obtained experimentally for the 5 sensitivity points of the sensor with Kapton substrate and with tattoo paper substrate (dashed circles indicates the sensitivity points).

Figure S-B4 shows the *n-type* and *p-type* signals of the e-tattoo when it is touched in the center of the configuration.


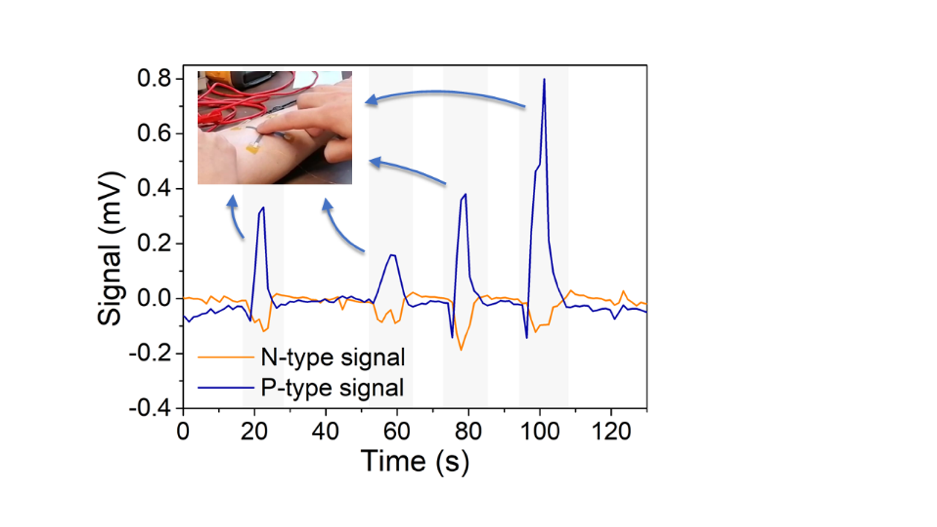


**Figure S-B4.** Sensor responses to the touch of a finger in the center of the configuration.

Figure S-B5 shows results related with the mechanical stability and resistance to the existence of artificial sweat on the surface of the e-tattoo. In Figure S-B5-(a) the e-tattoo is compressed and stretched in the skin and the resistance increase up to 5% the initial value, decreasing after the deformation and maintaining the initial performance. In Figure S-B5-(b) it is represented a test to the mechanical stability of the stripes when printed in tattoo paper and with the layer that stays between the sensor and the skin. This study shows an increase on the resistance in the moment of higher curvature (curvature radius of 5.6mm), but with and decrease when the sample comes back to the normal position. After some cycles the stripes acquired some flexibility, with lower resistance variations. In Figure S-B5-(c) and (d) it is represented the individually variation of the stripes resistance with the application of sweat. When the sweat is spray on the stripe, the resistance increase, however, after some time, the sweat evaporates and the resistance returns to the initial value.


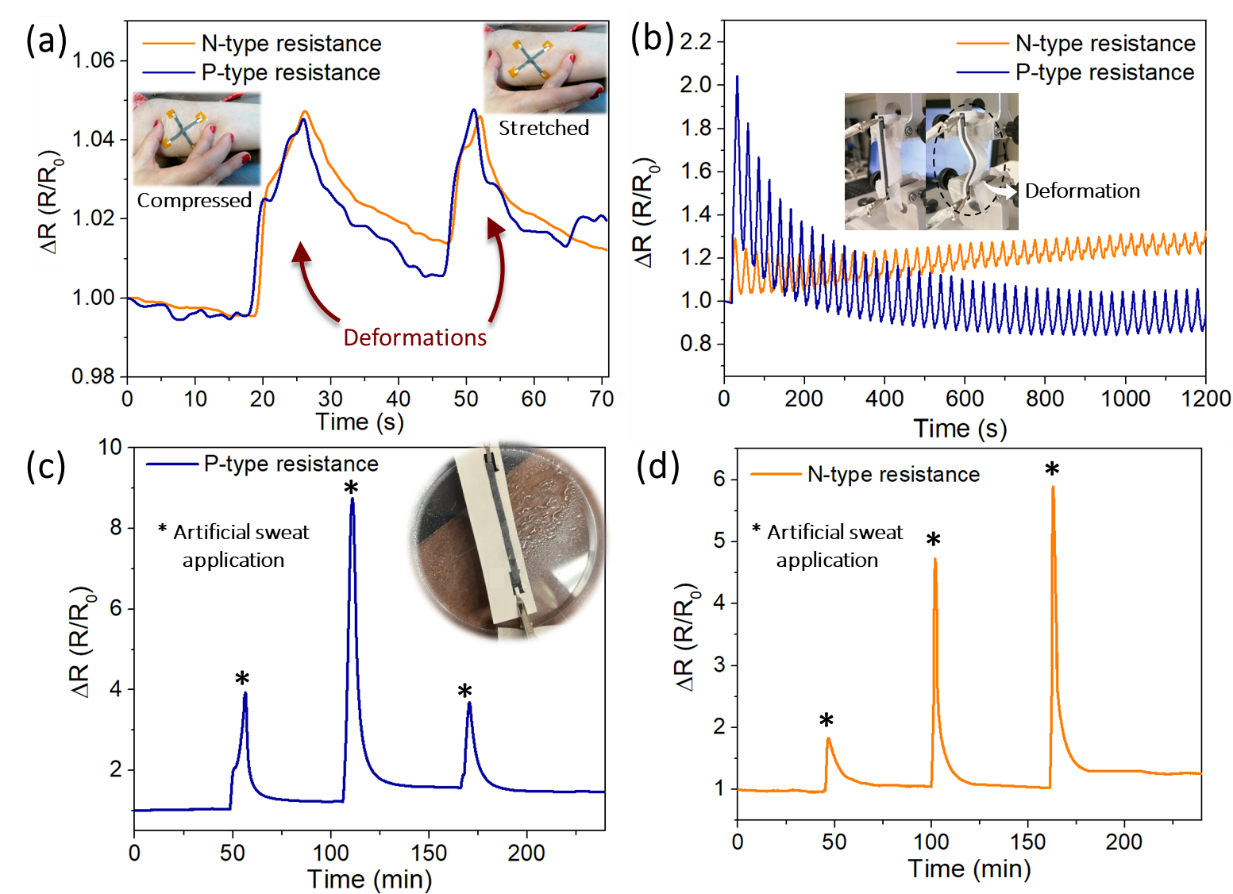


**Figure S-B5.** (a) Resistance variation of each stripe of e-tattoo when a deformation is applied, namely compression and stretching as indicated in the inset images. (b) Test to the mechanical stability of the stripes printed in tattoo paper over several cycles with a curvature radius of 5.6mm. (c) Variation of resistance of p-type stripe and (d) n-type stipe with the application of sweat.

Figure S-B6 shows the numerical results that proves the possibility of expanding the configuration presented in this work in order to increase the ratio between sensitivity points and measured signals. In this configuration with three stripes of a *p-type* material and *n-type* material it is possible to analyze 21 points of the grid only with 6 signals. The point of temperature change will be the point that corresponds to the interception of the two stripes with higher signal. This ratio can be important in large surface area monitoring, reducing the number of contacts and data to be analyzed.


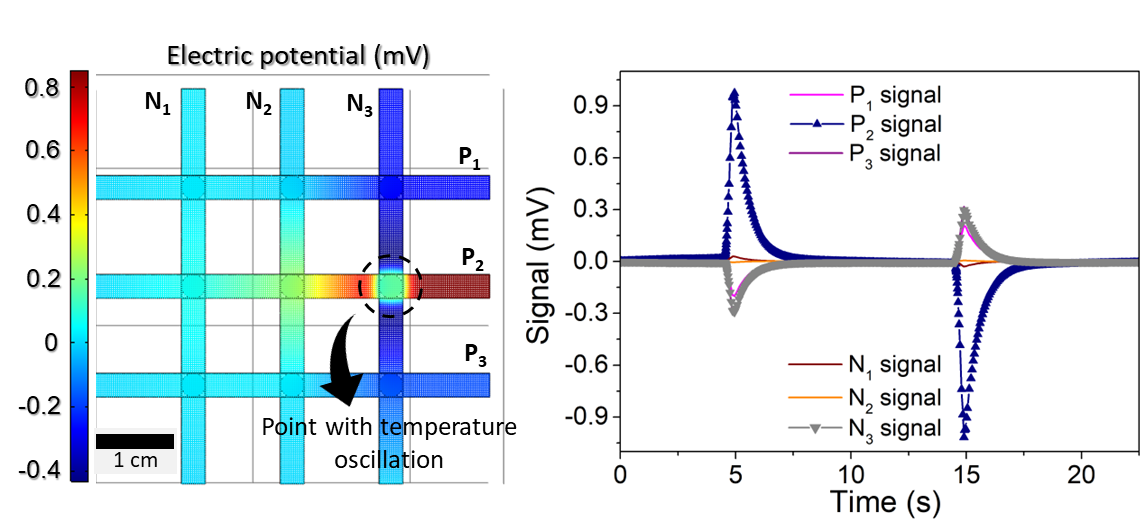


**Figure S-B6.** Numerical results of a configuration with three n-type and p-type stripes. The point with temperature variation can be identified by the interception of the stripes with higher signals.

Figure S-B7 depicts the flexible printed film production method employed in this study. The process begins with weighting the different components – polymers and Inorganic powder - (see Figure S-B7-(a-i)). Then, a homogeneous thermoelectric ink is formulated through constant and vigorous stirring (see Figure S-B7-(a-ii)). To print the films and devices, a PET 1500 27/120W mesh with specific designs was used, where the ink is pushed through the mesh’s pores using a squeegee (see Figure S-B7-(a-iii)). This technique involves the direct contact transfer of ink from a stenciled mesh to a target substrate, namely Kapton and/or tattoo paper. After drying, homogeneous printed thermoelectric films are obtained (see Figure S-B7-(a-iv)). A M-PRINT manual screen printing machine was employed for producing the printed thermoelectric films and the thermoelectric devices - Figure S-B7-(b) - and is available at the *Institute of Physics for Advanced Materials, Nanotechnology and Photonics* (IFIMUP) facilities.


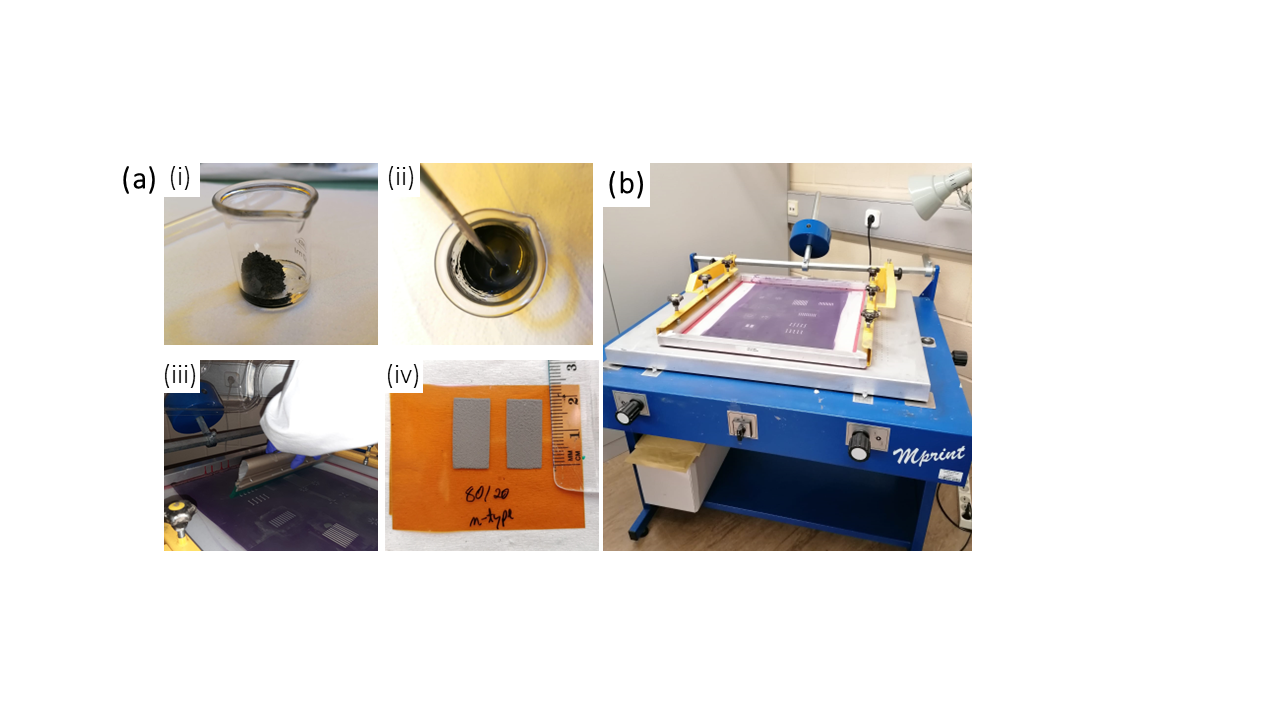


**Figure S-B7.** (a) Steps implemented to produce the screen-printed films: (i) Weighing the materials (powder and polymer), (ii) Stirring the mixture to form a homogeneous thermoelectric ink, (iii) Placing the ink on the screen and subsequent printing of the films using a squeegee, (iv) Resulting printed thermoelectric films on Kapton substrate. (b) Screen-printing equipment used in this work.

Figure S-B8 represents the experimental setup used to characterize the developed sensors. It uses a *Keithley 2182* nanovoltmeter connected to a LabView routine in the computer to analyze the generated signals over time, a *DC Current and Voltage Calibrator* from *Time Electronics* to power the Peltier module, a *PicoLog* data logger connected to a Type K thermocouple to monitor the temperature gradients in the sensors. The Peltier module, thermocouples and the sensor are placed above a heatsink. In this figure, it is also possible to observe one real image of this setup.


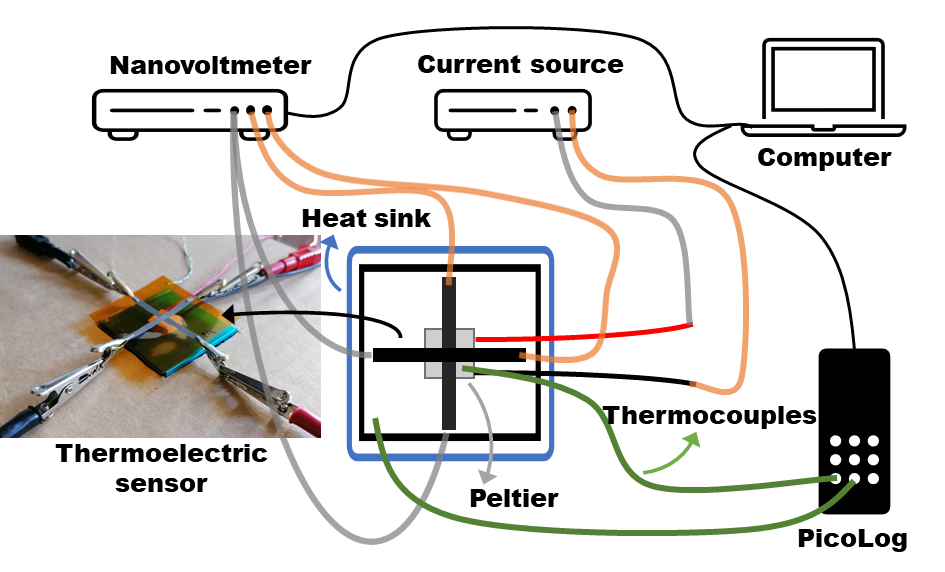


**Figure S-B8**. Experimental setup used for sensor calibration. This setup uses a heatsink and a Peltier module under the sensor, a power supply to control the Peltier module, two thermocouples connected to PicoLog to control the temperature gradients, a nanovoltmeter to read the signals and a computer for acquisition of experimental data.

1. **Corresponding authors:**

   André M. Pereira^#^,

   Email: [ampereira@fc.up.pt](mailto:ampereira@fc.up.pt), FCUP-IFIMUP

   Ana L. Pires^*^,

   Email: [ana.pires@fc.up.pt](mailto:ana.pires@fc.up.pt), FCUP-IFIMUP [↑](#footnote-ref-1)
